# Salt gradient-driven adaptation in okra: uncovering mechanisms of tolerance and growth regulation

**DOI:** 10.3389/fpls.2025.1648092

**Published:** 2025-07-23

**Authors:** Xi Yang, Jiuxing He, Lifeng Xu, Meng Kong, Qiuyan Huo, Jiqing Song, Wei Han, Guohua Lv

**Affiliations:** ^1^ Institute of Environment and Sustainable Development in Agriculture, Chinese Academy of Agricultural Sciences, Beijing, China; ^2^ Xianghu Laboratory, Hangzhou, China; ^3^ Mihe National Wetland Park Management Service Center, Qingzhou, China; ^4^ Shandong Agri-tech Extension Center, Jinan, China; ^5^ State Key Laboratory of Efficient Utilization of Agricultural Water Resources, China Agricultural University/Chinese Academy of Agricultural Sciences (CAU/CAAS), Beijing, China

**Keywords:** okra, salt tolerance, ion homeostasis, transcriptomics, photoprotection key

## Abstract

Soil salinity is an increasingly critical constraint on crop establishment and yield stability, especially in marginal and irrigated agricultural zones. Despite its nutritional and economic value, the mechanistic basis of salt tolerance in *Abelmoschus esculentus* (okra) remains poorly defined. Here, we integrated physiological phenotyping with transcriptome profiling to elucidate the stage-specific strategies employed by okra in response to NaCl stress. Our results revealed a bifurcated salt response: germination was highly sensitive, with complete inhibition at ≥ 0.5% NaCl, whereas seedling growth exhibited a hormetic pattern, being promoted under mild salinity (0.1–0.3%) and suppressed at higher levels. Photosynthetic integrity and photoprotection were preserved under low salinity but declined under severe stress, accompanied by increased oxidative burden. Transcriptomic analyses revealed that moderate salt stress elicited the coordinated activation of ion homeostasis genes, calcium signaling components, and GH3-family auxin-responsive genes (log_2_FC = 2.3–2.5), suggesting a critical role for dynamic auxin conjugation in growth maintenance under ionic stress. Concurrently, ROS detoxification, cytoskeletal remodeling, and metabolic adjustments were induced to support cellular stability. These findings defines okra’s salt tolerance threshold, reveals key molecular targets for genetic improvement, and provides a scientific foundation for the sustainable deployment of salt-tolerant okra in saline agriculture and land reclamation.

## 1 Introduction

Soil salinization is a major environmental challenge that threatens global agricultural productivity, especially in arid and semi-arid regions, where intensive irrigation practices exacerbate salinity levels. Currently, around 20% of the world’s arable land is affected by salinity, leading to substantial reductions in crop yields and food security (Hassani et al., 2021; Muhammad et al., 2024; Mukhopadhyay et al., 2021). In China, regions such as the Yellow River Delta and the Northeast Plain are severly affected by salinization due to excessive irrigation and poor drainage, highlighting the urgent need for sustainable water management strategies (Haj-Amor et al., 2022; Zhou et al., 2023). As climate change accelerates soil salinization, the development of salt-tolerant crops is crucial for ensuring agricultural sustainability and water use efficiency in vulnerable regions (Munns, 2002; Rozema and Flowers, 2008).

Okra (*Abelmoschus esculentus*), a drought-tolerant, high-value crop, is widely cultivated in saline-prone regions of Africa, Asia, and the Mediterranean (Elkhalifa et al., 2021; Kamaluldeen et al., 2014). Its ability to thrive in challenging environments makes it an important crop for ensuring food security and agricultural sustainability in areas affected by soil salinization. However, the molecular and physiological mechanisms underlying its salt tolerance remain inadequately explored, especially when compared to model species such as *Arabidopsis thaliana* and major crops like rice, wheat, and barley, for which significant progress has been made in understanding salt stress responses (Hu and Schmidhalter, 2023; Melino and Tester, 2023; Y. Zhang et al., 2023). A more comprehensive understanding of these mechanisms in okra could offer valuable insights for enhancing its productivity and resilience under saline conditions, contributing to more efficient water use and sustainable agricultural practices.

A critical aspect of salt tolerance is maintaining of an optimal Na^+^/K^+^ ratio, as excessive Na^+^ accumulation in the cytoplasm leads to cellular toxicity (Ji et al., 2013; Munns and Tester, 2008). Plants have evolved sophisticated strategies to mitigate this, including the compartmentalization of Na^+^ into vacuoles and its active exclusion via ion transporters such as high-affinity K+ transporters (HKT1), sodium-hydrogen exchanger (NHX1), and salt overly sensitive (SOS1) (Bassil et al., 2011; Madrid-Espinoza et al., 2025; Rao et al., 2021; X.-y. Zhang et al., 2023). Additionally, the accumulation of compatible solutes (e.g., proline, glycine betaine, and sugars) plays a vital role in osmotic adjustment and the protection of cellular macromolecules under salinity (Chourasia et al., 2021; Zhou et al., 2024). Salt stress also induces the production of reactive oxygen species (ROS), which damage cellular structures and require robust antioxidant defense systems involving enzymes. such as superoxide dismutase (SOD), catalase (CAT), and peroxidases (POD) (ElSayed et al., 2021; Mani et al., 2024).

Transcription factors (TFs) play a critical role in regulating plant responses to salt stress by orchestrating complex gene expression networks that modulate ion transport, osmotic homeostasis, and stress signaling. The roles of WRKY, DREB (dehydration-responsive-element-binding), and AP2/ERF families in mediating salt stress responses have been widely studied(X. Zhu et al., 2025). MYB, bHLH, and ICE1-CBF/DREB1 families also contribute significantly to abiotic stress adaptation. For example, MbMYBC1 and MbICE1 from *Malus baccata* have been shown to confer enhanced salt and cold tolerance through transcriptional regulation of stress-responsive genes (Duan et al., 2022; Liu et al., 2023). Similarly, MxWRKY53 and MxNAS3 from *Malus xiaojinensis* are implicated in Na^+^/K^+^ homeostasis and ROS scavenging under salt stress conditions (Han et al., 2018; Jiang et al., 2014), further underscoring the complexity and functional diversity of TF-mediated regulation. Furthermore, key signaling pathways mediated by plant hormones (e.g., ABA, auxin, and ethylene) and kinases (e.g., CDPKs and MAPKs) play essential roles in regulating salt stress responses, reinforcing the importance of hormonal regulation in plant stress adaptation (Chen et al., 2021; Waadt et al., 2022). While significant progress has been made in understanding the physiological and molecular mechanisms of salt tolerance, much of this research has been conducted under short-term salt stress conditions applied after plants have been established under optimal growth conditions (Guo et al., 2024; Shi and Gu, 2020; Wang et al., 2023; Wu et al., 2021; Y. Zhang et al., 2023). However, this approach does not fully reflect the prolonged, continuous salt exposure that plants encounter in natural saline environments, particularly during early growth stages (Saleem et al., 2011; Zhan et al., 2019; Zhao et al., 2022).

By simulating realistic saline soil conditions, this study systematically investigated okra’s responses to sustained salt stress from germination through early seedling development. Integrated physiological and transcriptomic analyses revealed the key adaptive mechanisms underlying early-stage salt tolerance in okra and identified candidate molecular targets for genetic improvement. Importantly, we define the physiological threshold of okra’s salt tolerance and provide mechanistic insights that may inform the breeding and deployment of salt-tolerant cultivars for use in marginal or salt-affected agroecosystems.

## 2 Results

### 2.1 Seed germination responses to salinity stress in okra

**Figure 1:**
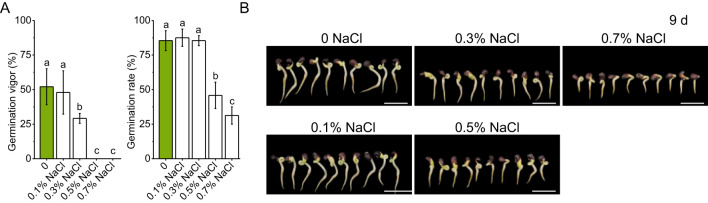
Germination of okra seeds under different salinity conditions. **(A)** Germination rates of okra seeds exposed to varying salt concentrations, with data presented as mean ± standard deviation. **(B)** Representative images of okra seedlings at day 9 of germination under different salinity treatments. The scale bar in each image represents 2 cm. Data were analyzed using one-way analysis of variance (ANOVA), followed by Duncan’s multiple range test for *post-hoc* multiple comparisons. Significant differences among groups (α = 0.05) are denoted by different lowercase letters (a, b, c,…), where groups sharing common letters indicate no statistically significant differences (*P* > 0.05). Values are expressed as mean ± SD with n ≥ 6 independent replicates.

Okra seeds exhibited distinct, dose-dependent germination responses to increasing NaCl concentrations (**Figure 1**). Germination vigor (Equation 1) declined significantly under moderate salinity, with a 44% reduction at 0.3% NaCl relative to the control), and was completely inhibited at concentrations ≥ 0.5% NaCl (**Figure 1A**). In contrast, the germination rate (Equation 2) remained stable under mild salinity, showing a slight but non-significant increase at 0.1%–0.3% NaCl. However, at 0.5% NaCl, the rate dropped sharply by 46.34%, and further declined to 31.25% at 0.7% NaCl, representing a 63.42% reduction compared to the control. These results suggest a physiological threshold for okra germination under low-grade salinity (0.1–0.3% NaCl), beyond which both vigor and rate decline rapidly, with complete inhibition occurring at ≥ 0.5% NaCl.

Phenotypic assessments of 9-day-old seedlings further substantiated these findings (**Figure 1B**). Under control and 0.1% NaCl conditions, radicle and plumule growth proceeded robustly, indicating minimal early developmental stress. At 0.3% and 0.5% NaCl, radicle elongation was partially inhibited, and plumule development became visibly stunted. Under 0.7% NaCl, both structures were severely impaired, with shortened radicles and arrested plumule growth. These morphological changes highlight the detrimental effects of moderate to high salinity on seedling establishment and early growth.

### 2.2 Phenotypic response of okra seedlings to salt stress

**Figure 2:**
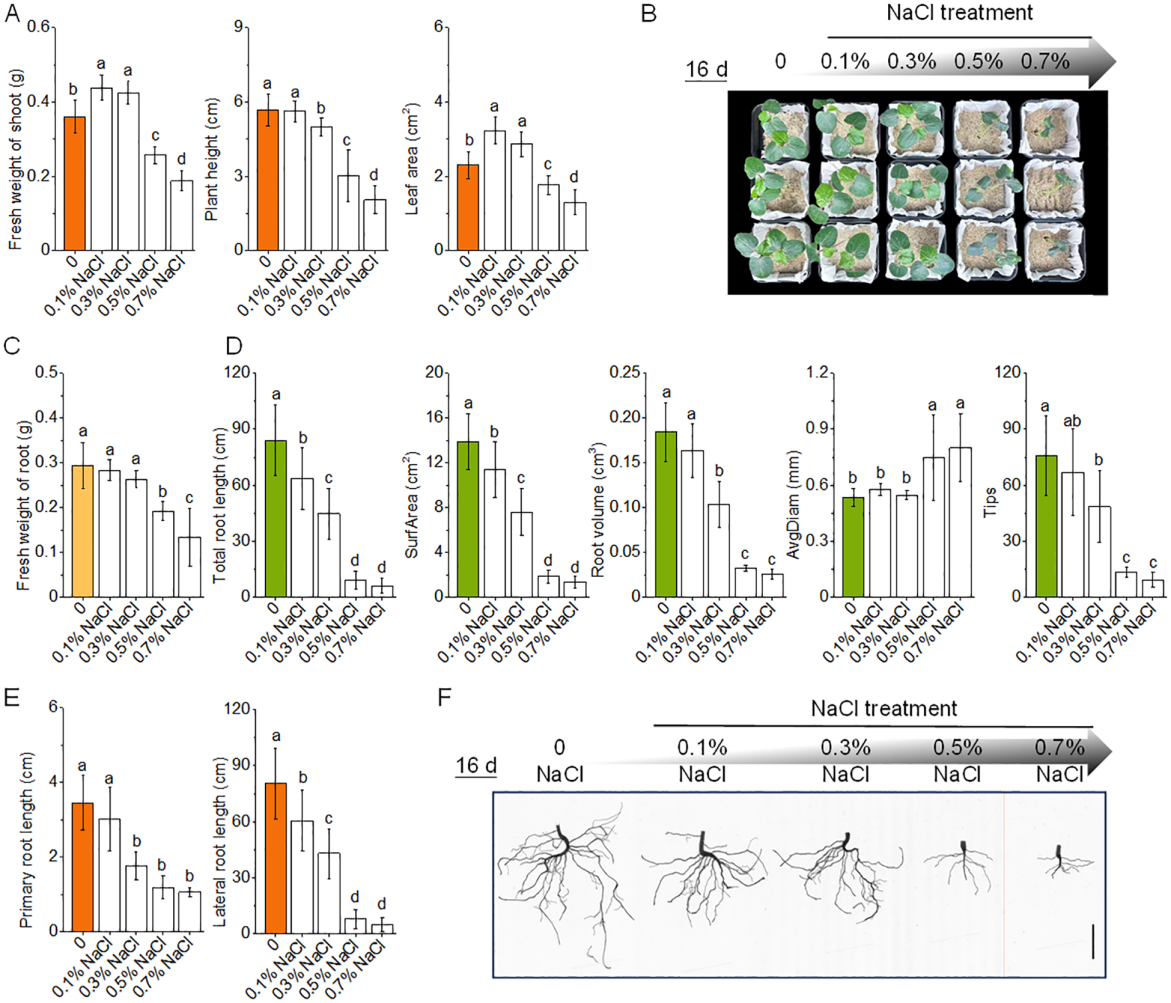
Effects of salinity stress on the growth and root morphology of okra seedlings. **(A)** Fresh weight of shoot, plant height, and leaf area of okra seedlings after 16 days of growth under varying NaCl concentrations (0%, 0.1%, 0.3%, 0.5%, and 0.7%). **(B)** Representative crown images of okra seedlings taken after 16 days of exposure to 0–0.7% NaCl treatments, illustrating the visual impact of salinity on shoot growth. **(C)** Fresh weight of roots of okra seedlings under different salinity conditions. **(D)** Root system traits, including total root length, total root surface area, total root volume, average root diameter, and root tip number, derived from root scanning analysis under different NaCl treatments. **(E)** Primary and lateral root lengths under varying NaCl concentrations, showing strong inhibition of lateral root development at higher salinity levels. **(F)** Representative root images of 16-day-old okra seedlings under NaCl treatments. The scale bar in each image represents 2 cm.

Okra seedlings exhibited a biphasic growth response to salt stress, with enhanced shoot performance under mild stress (0.1–0.3% NaCl) and marked inhibition at higher concentrations (≥ 0.5% NaCl) (**Figures 2A, B**). At 0.1% NaCl, shoot biomass increased by 21.8%, accompanied by a 39.9% expansion in leaf area relative to control (0% NaCl), consistent with a hormetic effect wherein low-level stress stimulates growth. In contrast, root traits such as total root length and volume showed only minor reductions (−24.2% and −11.5%, respectively), suggesting that root functionality remained largely preserved under subtoxic salinity (**Figures 2C–F**).

Beyond 0.3% NaCl, salinity induced progressive inhibition of shoot and root development. Shoot biomass declined by 28.7% and 47.8% at 0.5% and 0.7% NaCl, respectively, while total root volume and root length decreased by 82.4% and 89.2%. A dramatic 87.7% reduction in root tip number at 0.7% NaCl indicated compromised rhizospheric exploration. Notably, root thickening was observed under salt stress, with root diameter increasing by 40.2–50.0%, reflecting an allometric shift potentially aimed at structural compensation. However, this hypertrophic adjustment coincided with significant losses in absorptive surface area (−86.7% to −90.3%), suggesting diminished functional efficiency. Together, these results define a physiological tipping point at 0.3% NaCl, below which mild salinity promotes growth, but above which developmental and architectural integrity become progressively compromised.

### 2.3 Photosynthetic performance of okra seedlings under salt stress

**Figure 3:**
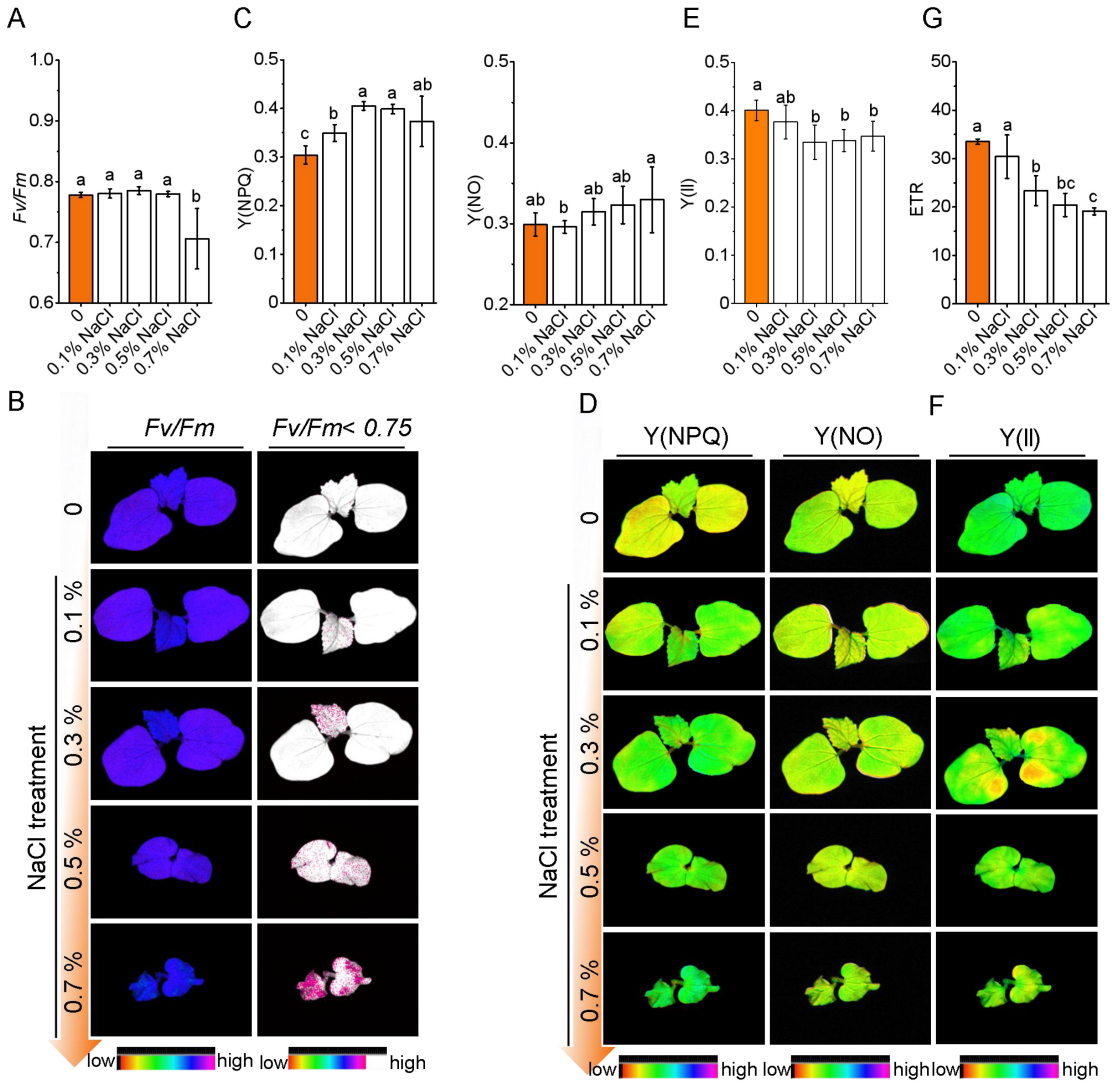
Photosynthetic response of okra seedlings to salt stress under varying NaCl concentrations. **(A)** *Fv/Fm* values of photosystem II (PSII) under varying NaCl concentrations (0%, 0.1%, 0.3%, 0.5%, and 0.7% NaCl). **(B)** Representative chlorophyll fluorescence images showing the effect of salt stress on PSII efficiency across different NaCl treatments. **(C)** Y(NPQ) and Y(NO) values indicating regulated energy dissipation and non-regulated energy dissipation. **(D)** Representative chlorophyll fluorescence images of okra seedlings showing PSII activity and energy dissipation at different NaCl concentrations. **(E, F)** Quantification and Representative chlorophyll fluorescence images of Y(II). **(G)** ETR values (electron transport rate) under different NaCl concentrations.

Okra seedlings exhibited a concentration-dependent photosynthetic response to salt stress. Under mild salinity (0.1–0.3% NaCl), the structural integrity of photosystem II (PSII) remained largely intact, as indicated by stable *Fv/Fm* values (within ± 3.2% of control) (**Figures 3A, B**). Chlorophyll fluorescence imaging showed spatially uniform PSII activity, consistent with the activation of systemic photoprotective responses. Notably, non-photochemical quenching [Y(NPQ)] increased significantly by 14.8%–33.1% under 0.1%–0.3% NaCl (*P*< 0.05), suggesting enhanced thermal energy dissipation (**Figures 3C, D**). While the quantum yield of PSII [Y(II)] remained unaffected at 0.1% NaCl, it declined by 16.5% under 0.3% NaCl (*P<* 0.01), accompanied by a consistent decrease in electron transport rate (ETR) (**Figures 3E–G**), suggesting the onset of functional PSII impaired at this threshold.

Under high salinity (≥ 0.5% NaCl), PSII activity was substantially compromised. *Fv/Fm* declined sharply (to 0.71 at 0.7% NaCl), and ETR decreased by 43.0%, reflecting impaired electron flow. Meanwhile, Y(NPQ) declined, and non-regulated energy dissipation [Y(NO)] increased significantly (by 10.3%), indicating a breakdown of protective mechanisms and accumulation of photodamage. Fluorescence imaging revealed spatial heterogeneity in *Fv/Fm*, with localized zones of reduced PSII efficiency and impaired energy dissipation capacity. Together, these findings suggest that okra maintains photochemical stability under mild salinity (≤ 0.1% NaCl) through photoprotective compensation. However, salinity levels ≥ 0.3% exceed the threshold of these protective responses, resulting in irreversible PSII damage and reduced photosynthetic efficiency.

### 2.4 Antioxidant dynamics and oxidative stress regulation under salinity

**Figure 4:**
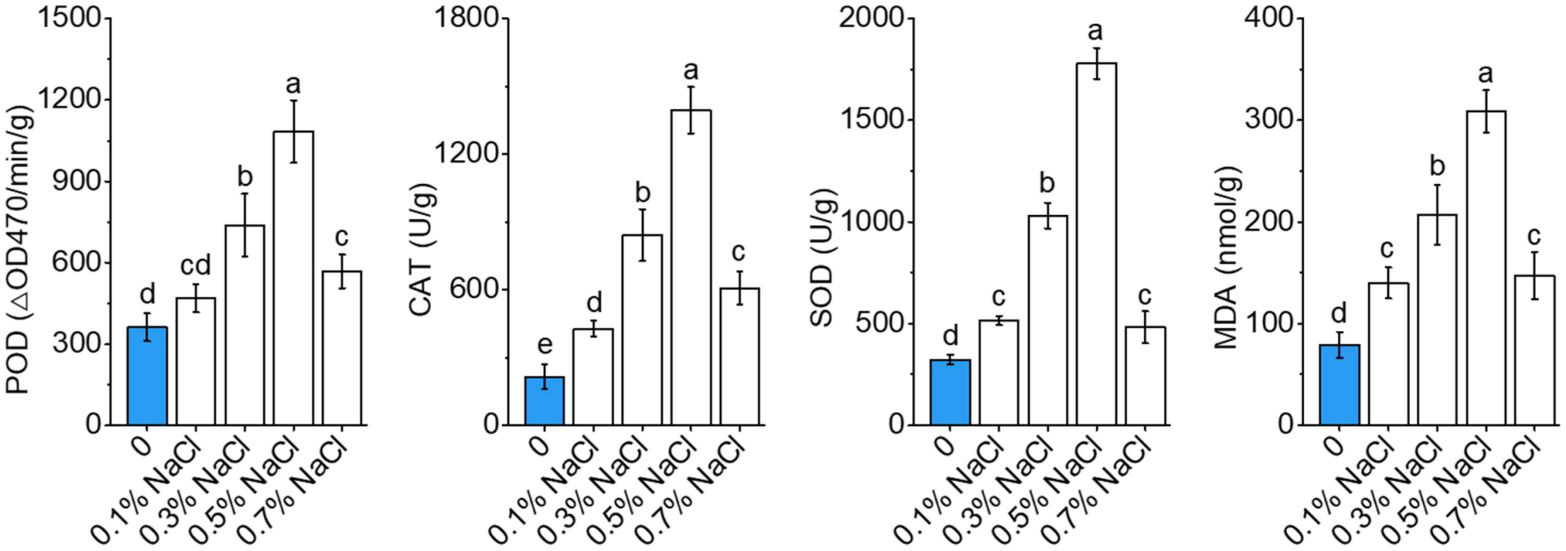
Antioxidant dynamics and oxidative stress regulation under salinity stress in okra seedlings. The activities of key antioxidant enzymes, including peroxidase (POD), catalase (CAT), and superoxide dismutase (SOD), as well as malondialdehyde (MDA) levels, under varying NaCl concentrations (0%, 0.1%, 0.3%, 0.5%, and 0.7% NaCl) after exposure to salt stress. Data are means ± SD.

Salt stress triggered dose-responsive modulation of antioxidant defenses and oxidative damage markers in okra seedlings (**Figure 4**). Progressive induction of core antioxidant enzymes (POD, CAT, and SOD) occurred under subtoxic to moderate salinity (0.1–0.5% NaCl; *P*< 0.01). POD activity reached maximum at 0.50% NaCl (1083.25 ΔOD 470/min/g; 3.0-fold elevation *vs*. control; *P*< 0.001), with parallel maxima for CAT (1394.42 U/g; 6.5-fold, *P<* 0.001) and SOD (1780.08 U/g; 5.5-fold, *P*< 0.01). These results indicate an enhanced antioxidant defense mechanism under moderate salt conditions to mitigate ROS and maintain cellular homeostasis. Supraoptimal salinity (0.70% NaCl) caused precipitous decline in enzyme activities (−47.6% POD; −56.4% CAT; −72.8% SOD; *P*< 0.05), concurrently with biphasic lipid peroxidation dynamics. MDA accumulation maximized at 0.50% NaCl (308.74 ± 24.13 nmol/g; +422% *vs*. control), followed by 18.3% reduction at 0.70% NaCl indicating catabolic failure or non-enzymatic peroxidation dominance. The 0.50% NaCl bifurcation point demarcates ROS homeostasis collapse when oxidative insult overwhelms detoxification capacity.

### 2.5 Transcriptomic plasticity reveals adaptive pathways under salt gradients

**Figure 5:**
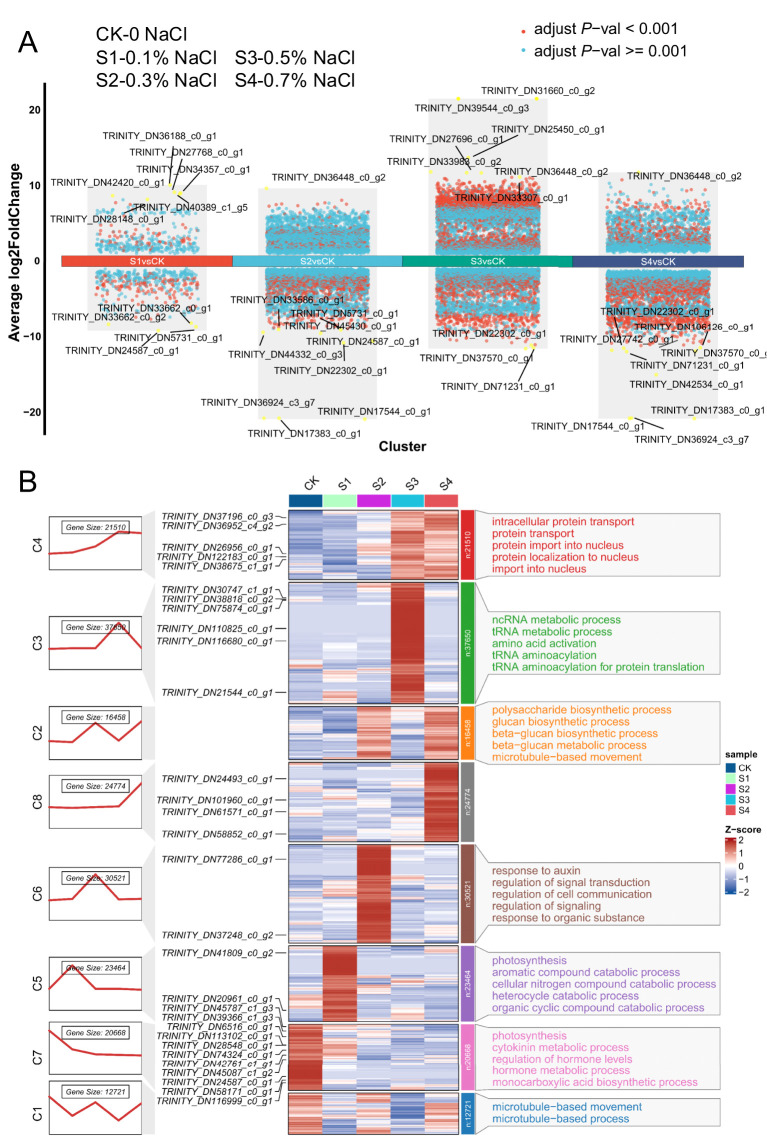
Global transcriptome analysis of okra seedlings under varying NaCl concentrations. **(A)** The Differential gene expression analysis showing up- and down-regulated genes across all ten clusters. An adjusted *P* value< 0.01 is indicated in red, while an adjusted *P* value ≥ 0.01 is indicated in black. **(B)** Hierarchical clustering analysis of gene expression patterns in okra seedlings under different NaCl treatments. Okra seeds were continuously exposed to either ddH_2_O (control) or NaCl concentrations ranging from 0.1% to 0.7%, and RNA samples were collected at 16 days of age for RNA-seq analysis. The figure presents (from left to right): gene expression trend fold plots, clustering analysis of differentially expressed genes (DEGs), and GO functional annotation (biological process, BP). Blue and red shading indicate down-regulated and up-regulated genes, respectively, highlighting significant expression changes in response to salinity stress.

To dissect transcriptional responses to salt stress, RNA-seq profiling was conducted on okra seedling leaves exposed to a salinity gradient (0.1–0.7% NaCl). The average Pearson’s correlation coefficient among the three biological replicates for each treatment exceeded 0.88, indicating high reproducibility and reliability of the RNA-seq dataset. Principal component analysis (PCA) clearly separated the nine samples into five distinct clusters corresponding to the different NaCl concentrations (0.1%–0.7%) and the control treatment (**Supplementary Figure S1**), reflecting substantial divergence in transcriptomic profiles among salinity conditions. A total of 17,736 differentially expressed genes (DEGs; FDR< 0.05) were identified, indicating extensive transcriptomic reprogramming (**Figure 5A**). The most pronounced response occurred at 0.5% NaCl, with 11,114 DEGs (7315 upregulated, 3829 downregulated), followed by 0.3% (6956 DEGs), 0.7% (6075 DEGs), and 0.1% NaCl (800 DEGs), suggesting a threshold-dependent transcriptional shift. Gene Ontology (GO) enrichment revealed distinct salt-responsive pathways (**Figure 5B**; **Supplementary Figure S2–S3**). Under mild salinity (0.1–0.3% NaCl), genes involved in microtubule organization and polysaccharide biosynthesis were upregulated, indicative of cytoskeletal remodeling and potential cell wall reinforcement. Photosynthesis-related genes, particularly those encoding thylakoid membrane components and photosystem subunits, were significantly induced at low salinity, consistent with enhanced photosynthetic capacity. Concurrently, genes related to mitochondrial function and redox balance were differentially expressed, suggesting adjustments in energy metabolism and ROS homeostasis. The upregulation of cytoskeletal motor proteins and protein-binding factors points to enhanced intracellular transport and structural maintenance under stress. Additionally, elevated expression of hexosyltransferases and UDP-glucosyltransferases implies increased glycoprotein and lipid modifications, potentially contributing to membrane stability and stress signaling. Collectively, these results demonstrate that salt stress induces a coordinated transcriptional program in okra seedlings, involving cytoskeletal dynamics, metabolic adjustment, and structural fortification to enhance resilience across a salinity gradient.

### 2.6 Key growth- and stress-responsive transcripts orchestrating salt adaptation

To delineate the molecular underpinnings of okra’s adaptive response to salinity, DEGs were categorized by function and pathway enrichment across salt treatments. At 0.1% NaCl, early transcriptional activation of oxidative stress-related genes (GO:0006979, *p* = 0.010982) was observed (**Supplementary Figure S4**; **Supplementary Tables S1–S2**). Several antioxidant genes—including *TRINITY_DN37402_c0_g3* (5.25-fold), *DN38873_c0_g1* (1.27-fold), *DN40545_c0_g1* (6.24-fold), and *DN40545_c0_g3* (5.53-fold)—were consistently upregulated across all salinity levels, suggesting their central role in ROS detoxification. At 0.3% NaCl, robust enrichment was detected for hormone response pathways (GO:0009725, GO:0009719; *P* ≤ 1.03 × 10^-10^), notably involving *GH3* family genes implicated in auxin conjugation. *TRINITY_DN42469_c0_g1*, *DN49280_c0_g2*, and *DN49280_c0_g1* exhibited log_2_ fold changes of 4.28, 1.36, and 3.69, respectively (**Supplementary Tables S3–S4**), indicating active auxin homeostasis reprogramming in response to moderate salt stress. At higher salinity (0.5%–0.7% NaCl), genes involved in defense response (GO:0006952) and oxidoreductase activity (GO:0016491) were significantly upregulated, peaking at 0.5% NaCl (*P* = 6.15 × 10^-^¹³). Enrichment of peroxide reduction (GO:0016684; *P* = 8.31 × 10^-7^) further highlighted enhanced redox homeostasis machinery under stress. However, at 0.7% NaCl, DEG enrichment sharply declined, suggesting a physiological threshold beyond which transcriptional plasticity is compromised.

Ion transport-related responses were strongly context-dependent. At 0.1% NaCl, pathways related to metal ion homeostasis (Fe, Mn) were activated, potentially supporting antioxidant enzyme function. At 0.3%, enrichment shifted toward organic anion transport, suggesting an osmotic adjustment strategy. At 0.5% NaCl, monovalent cation transport (Na^+^/K^+^) was preferentially upregulated, indicating ion homeostasis efforts via selective Na^+^ exclusion and K^+^ retention. In contrast, no ion transport pathways were enriched at 0.7% NaCl, implying functional exhaustion of ion regulatory capacity. Calcium signaling genes exhibited dynamic, stress-dependent activation. Enrichment of calcium-binding functions (GO:0005509; *P* = 0.00017) peaked at 0.3% NaCl, underscoring the role of Ca²^+^-mediated signaling in stress perception. Notably, *TRINITY_DN51471_c1_g2* responded specifically to 0.1% NaCl, while *DN126826_c0_g1* and *DN37569_c0_g1* were consistently upregulated at 0.3–0.7% NaCl, indicating a shift in calcium signaling effectors across stress intensities.

**Figure 6:**
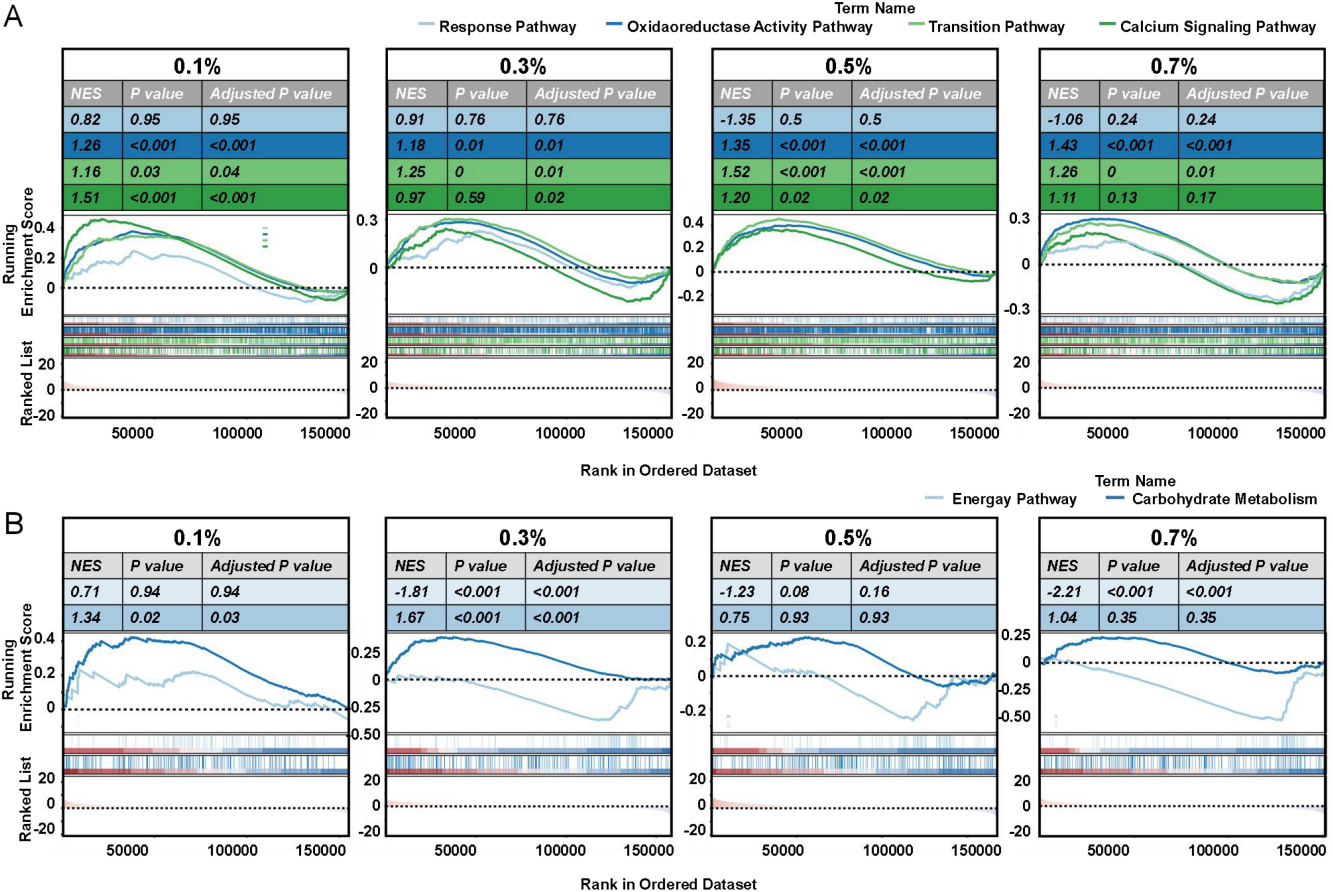
GSEA analysis of key pathways in okra seedlings under varying NaCl treatments. Gene set enrichment analysis (GSEA) was performed to identify significant pathways in okra seedlings exposed to different NaCl concentrations (0%, 0.1%, 0.3%, 0.5%, and 0.7% NaCl). Pathways analyzed include **(A)** response pathway, oxidoreductase activity pathway, ion transition pathway, calcium signaling pathway, **(B)** energy pathway, and carbohydrate metabolism pathway. For each pathway, the plot displays normalized enrichment scores (NES), *P* values, and adjusted *P*-values, with blue and red shading indicating down- and up-regulated gene sets, respectively. Each line in the plot represents a gene’s rank within the gene set.

Gene set enrichment analysis (GSEA) reinforced these findings (**Figure 6**). Oxidoreductase pathways peaked at 0.3–0.5% NaCl, declining at 0.7%, indicating a collapse of detoxification capacity under extreme stress. Calcium signaling was most active at lower salinities (0.1–0.3%), reflecting early stress perception, but diminished at higher stress, likely due to receptor desensitization or energy reallocation. Ion transport activity fluctuated across treatments, reflecting sustained efforts to stabilize ionic equilibrium. In terms of metabolism, energy production pathways were initially suppressed (0.1%) but upregulated at 0.3% NaCl, supporting stress-induced ATP demand (**Supplementary Table S5**). This activation declined under high salinity, likely reflecting metabolic fatigue. In contrast, carbohydrate metabolism was progressively suppressed, reaching a minimum at 0.7% NaCl (**Supplementary Table S6**), consistent with resource reallocation toward defense pathways or disruption of central metabolic processes under severe stress.

## 3 Discussion

**Figure 7:**
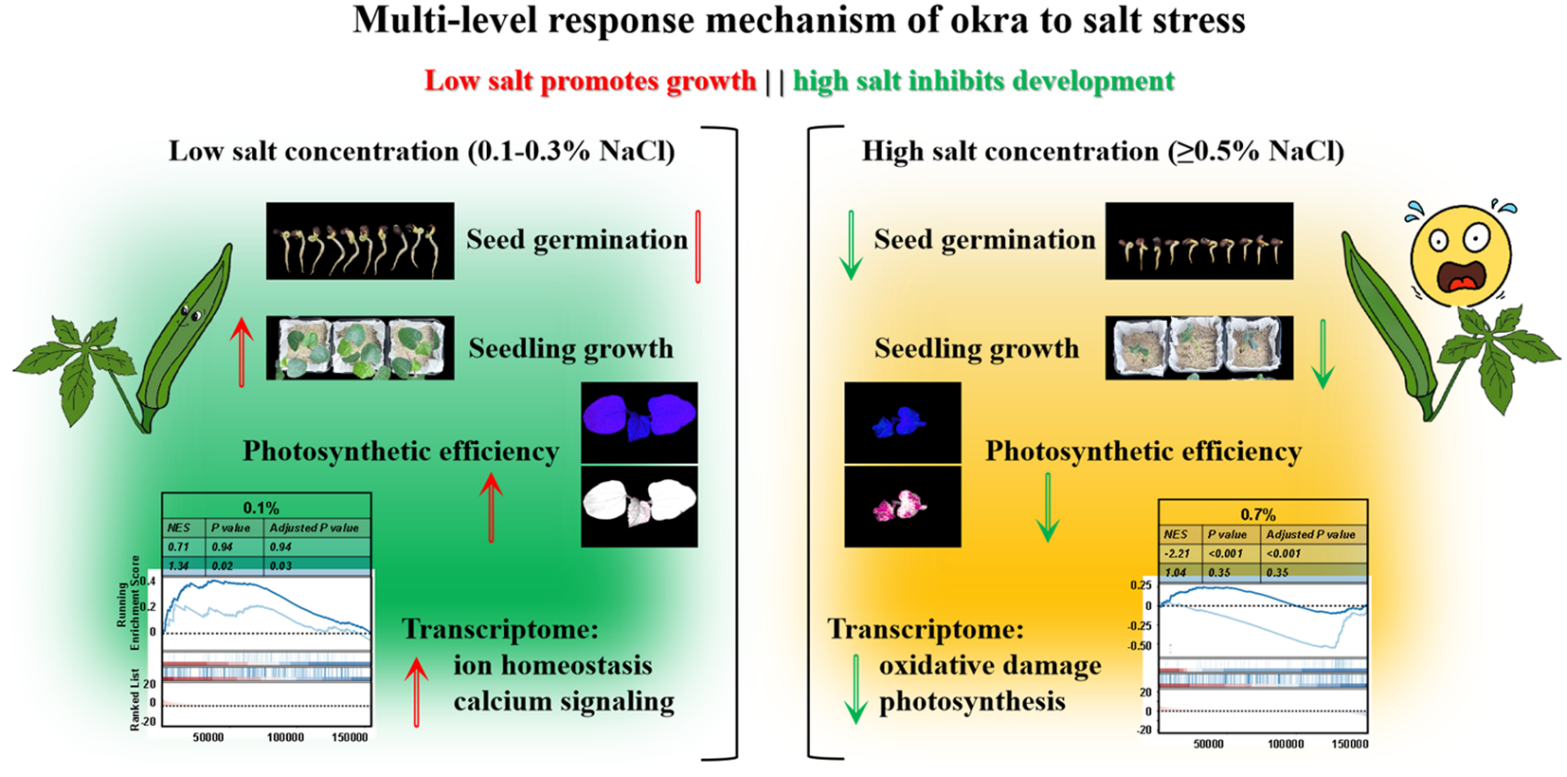
Dual-phase model of salt stress response in okra (*Abelmoschus esculentus*). At low salinity (0.1–0.3% NaCl), ROS–calcium signaling initiates antioxidant defenses (e.g., SOD, CAT) and ion homeostasis mechanisms, synergistically enhancing photosynthetic performance and promoting adaptive root remodeling. These responses collectively support continued growth under mild stress. In contrast, high salinity (> 0.3% NaCl) induces a systemic breakdown characterized by antioxidant system overload (as indicated by MDA accumulation), Na^+^/K^+^ imbalance, and impaired energy metabolism, ultimately resulting in photosynthetic damage and root functional decline.

Okra (*Abelmoschus esculentus*) displays a distinct biphasic dose–response to increasing salinity, with approximately 0.3% NaCl (~50 mM) emerging as a physiological threshold separating adaptive growth from stress-induced collapse (**Figure 7**). At mild salinity levels (0.1–0.3% NaCl, ~17–50 mM), okra exhibits either neutral or modestly growth-promoting effects, consistent with a hormetic response wherein sublethal stress primes physiological adaptation (Agathokleous, 2021). Such exposure likely initiates acclimatory processes that enhance biomass accumulation and photosynthetic efficiency, a pattern also observed in halophytic species (**Figures 2A, B**). For instance, *Limonium bicolor* demonstrates increased growth and antioxidant activity at ~100 mM NaCl, followed by decline at higher concentrations (Z. Zhu et al., 2025). Similarly, in okra, growth and developmental traits are sustained or modestly improved up to ~0.3% NaCl, supporting this concentration as a critical tipping point (**Figures 2**, **3**). Beyond this threshold (≥ 0.5% NaCl or ~85 mM), plants enter a decompensatory phase marked by pronounced growth inhibition, tissue damage, and systemic physiological dysfunction. These findings are consistent with germination data: okra seeds fail to germinate at ≥ 100 mM NaCl, whereas moderate salinity (25–50 mM) is partially tolerated, albeit with reduced germination rates (**Figure 1**) (Saima et al., 2022). Collectively, these results delineate a biphasic salt-response profile in okra, comprising an “acclimation zone” under mild salinity and a collapse phase triggered upon exceeding homeostatic limits. This pattern typifies stress-induced hormesis, wherein low salt levels elicit adaptive benefits, but surpassing critical thresholds in ionic and osmotic regulation leads to systemic failure.

The contrasting outcomes at mild and high salinity are underpinned by distinct physiological and molecular responses. Under mild salt stress, okra initiates a coordinated activation of defense and acclimation pathways that collectively mitigate damage and sustain growth. A moderate elevation in reactive oxygen species (ROS) under low salinity likely functions as a signaling cue to activate antioxidant systems and stress-responsive gene networks. This transient and controlled ROS burst can stimulate antioxidant enzymes—including SOD, CAT, and ascorbate peroxidase (APX)—as well as redox-sensitive transcription factors, thereby priming the plant’s defenses (**Figure 4**; **Supplementary Figure S4**; **Supplementary Tables S1–S2**) (Van Zelm et al., 2020). Indeed, *L. bicolor* seedlings under low salinity exhibit increased activities of ROS-scavenging enzymes during the early adaptation phase, and okra is presumed to employ a similar antioxidant strategy(Z. Zhu et al., 2025). Elevated levels of SOD, CAT, and POD have been reported in okra and other crops exposed to ~50 mM NaCl, indicating the mobilization of enzymatic ROS detoxification mechanisms (Naseem et al., 2023). These antioxidant defenses neutralize ROS and protect macromolecules, thereby safeguarding cells from oxidative injury under manageable stress levels. In contrast, at high salinity, ROS production exceeds the detoxification capacity, leading to uncontrolled oxidative stress and irreversible cellular damage. Salinity-induced oxidative stress is a canonical secondary effect of ionic toxicity, and can irreparably impair cell function when left unchecked (Das et al., 2024). In okra exposed to ≥ 0.5% NaCl, excessive Na^+^ accumulation and resultant ionic disequilibrium likely provoke excess ROS production, for example via perturbations in photosynthetic electron transport and NADPH oxidase hyperactivation, culminating in lipid peroxidation and membrane disruption (**Figure 3**). The accumulation of MDA—a well-established marker of lipid peroxidation—in severely salt-stressed okra further corroborates the occurrence of oxidative membrane damage (**Figure 4**) (Guo et al., 2025). Such oxidative injury impairs membrane integrity, resulting in electrolyte leakage and tissue necrosis. Thus, while mild salinity triggers ROS as signaling molecules within a controlled redox framework, high salinity elicits ROS as cytotoxic agents once antioxidant defenses are overwhelmed (Mittler et al., 2022).

Ion homeostasis is a central component of okra’s adaptive response to mild salinity. Like other moderately salt-tolerant glycophytes, okra likely engages conserved pathways—most notably the Salt Overly Sensitive (SOS) signaling cascade—to regulate Na^+^ and K^+^ balance under stress (Ji et al., 2013). Salt-induced cytosolic Ca^2+^ elevations activate the SOS pathway, wherein SOS3 (CBL4) and SOS2 (CIPK24) stimulate the plasma membrane Na^+^/H^+^ antiporter SOS1 to extrude Na^+^ from root cells (Ramakrishna et al., 2025; Xie et al., 2022). Concurrently, HKT transporters in the root xylem parenchyma retrieve Na^+^ from the xylem sap, limiting its translocation to aerial tissues and protecting photosynthetic organs from Na^+^ toxicity (Kronzucker and Britto, 2011). Under mild salt exposure (e.g. 25–50 mM), okra can likely sustain a high cytosolic K^+^/Na^+^ ratio by these means – extruding Na^+^ at roots, restricting its translocation to shoots, and storing excess Na^+^ in vacuoles (**Supplementary Table S1**, **Supplementary Table S2**) (Hauser and Horie, 2010). This ion-homeostatic control averts the disruption of metabolic processes that excessive Na^+^ causes. At higher salinities, however, these systems reach capacity or incur regulatory dysfunction. Indeed, severe salt stress (such as 100–150 mM NaCl) inundates the plant with Na^+^ faster than it can be extruded or compartmentalized (Almeida et al., 2017). The result is cytosolic Na^+^ accumulation, K^+^ loss, and enzyme inhibition, which together lead to metabolic collapse. Okra’s ability to delay this outcome up to ~0.3% NaCl reflects a moderately effective ion regulation system – stronger than that of highly sensitive species like rice, but obviously weaker than in true halophytes that can endure NaCl above 200 mM through specialized mechanisms (Grigore and Vicente, 2023; Wei et al., 2025).

Hormonal signaling adjustments also underlie okra’s biphasic salt response, particularly involving auxin dynamics. Under stress conditions, plants often redirect hormonal cues to favor survival over growth. In okra, the transcriptomic data highlighted GH3 gene induction, suggesting modulation of auxin homeostasis. GH3 genes encode auxin-conjugating enzymes (IAA-amido synthetases) that bind excess indole-3-acetic acid (IAA) to amino acids, thereby lowering free auxin levels and attenuating auxin signaling (**Supplementary Tables S3**, **S4**) (Singh et al., 2015). Many GH3 family members are known to be salt-responsive; for instance, several GH3 genes in legumes and *Arabidopsis* are strongly upregulated by NaCl stress (Park et al., 2007). The upregulation of GH3 in salt-treated okra implies that auxin inactivation is occurring as a stress adaptation. By curbing auxin, the plant likely suppresses organ growth (e.g. halting cell expansion and lateral root emergence)—a strategic reallocation of resources under stress (Zhang et al., 2009). This aligns with the commonly observed reduction in shoot growth and partial root growth inhibition under salinity, mediated by stress hormones (like abscisic acid and ethylene) and auxin crosstalk (Shani et al., 2017). In mild salinity, a controlled increase of GH3 and related auxin regulators can moderate growth just enough to prioritize defense, without completely stunting the plant. Conversely, at high salinity, hormonal balances may shift drastically. Abscisic acid (ABA) levels surge (due to osmotic stress), cytokinins decline, and ethylene production increases, collectively enforcing growth arrest (Ryu and Cho, 2015). Okra’s GH3-mediated auxin dampening under salt suggests an attempt to tune down growth-promoting signals in favor of stress tolerance signals. However, in extreme salinity this shift likely becomes extreme growth suppression (as seen by severe stunting and lack of new organ development at ≥0.5% NaCl).

Okra’s adaptive strategy under salt stress involves coordinated root morphological reprogramming coupled with biochemical adjustments. Salt stress modifies root system architecture, promoting the development of thicker primary roots while simultaneously suppressing fine root proliferation and reducing root hair length (**Figures 2C-F**) (Karlova et al., 2021). These changes, also documented in other crops such as wheat, represent a strategic trade-off: enhanced root barriers limit ion influx and water loss but reduce the absorptive surface area, thereby constraining nutrient and water uptake (Robin et al., 2016). Under mild salinity (0.1–0.3% NaCl), this trade-off appears sustainable—okra retains sufficient fine roots while fortifying its structural defenses. We also observed enhanced lateral root initiation under moderate salt conditions, likely as a compensatory response to decreased root efficiency—a pattern consistent with salinity-induced promotion of root branching and inhibition of root elongation (Ogorek et al., 2023). As salinity intensifies, resource allocation shifts further toward structural protection. Earlier and more extensive development of exodermal and endodermal barriers, accompanied by suppressed root hair formation, progressively diminishes the plant’s ability to absorb water and nutrients—even in moist soils—leading to visible shoot-level symptoms such as chlorosis and wilting (Acosta-Motos et al., 2017). Concomitantly, resource partitioning under salt stress is reoriented (**Figures 5**, **6**; **Supplementary Tables S5**, **S6**). Okra diverts metabolic assimilates away from growth toward cellular maintenance and defense. Energetically demanding processes—such as cell division and expansion—are downregulated, while carbon flux is redirected to support ion transport, compatible solute biosynthesis (e.g., proline, soluble sugars), and antioxidant metabolism (e.g., flavonoids, phenolics) (Ayub et al., 2020). Although this reallocation stabilizes intracellular conditions, it also contributes to growth retardation and reproductive impairment—manifesting as reduced harvest index, flower and fruit abortion, and overall yield decline (Zörb et al., 2019).

Ecologically, this represents a salt tolerance strategy rather than avoidance. Unlike halophytes, okra does not possess salt-exclusion mechanisms, but instead enhances internal defense systems to endure moderate salinity. For example, root suberization serves as a physical barrier conferring broad-spectrum stress tolerance—albeit at the expense of nutrient acquisition efficiency (Chen et al., 2025; Lu et al., 2022). In agricultural settings, these trade-offs carry critical implications. Agronomic practices such as maintaining optimal soil moisture or applying foliar nutrient supplements may mitigate the effects of reduced root uptake. Meanwhile, breeding strategies may target genotypes with minimal architectural penalties—such as those retaining finer roots or longer root hairs under saline conditions—to preserve productivity. Collectively, okra’s salt tolerance relies on a coordinated “ROS–ion–hormone–architecture” axis. Below 0.3% NaCl, oxidative signaling, hormonal modulation, and metabolic plasticity sustain growth; above this threshold, physiological damage overtakes transcriptional adaptation. This biphasic strategy sets okra apart from more salt-sensitive crops like rice. Moving forward, incorporating genotype–environment interactions and multi-omics analyses may help identify key regulators such as GH3s, and CDPKs. These findings may inform future efforts to improve okra’s resilience and productivity under saline conditions, particularly in environmentally constrained agricultural systems.

## 4 Materials and methods

### 4.1 Plant material and reagents

Experiments were conducted using the red-podded okra cultivar ‘*Meiren Zhi*,’ purchased from Beijing Fengming Yashi Technology Development Co., Ltd. Sodium chloride (NaCl) of analytical grade was obtained from Shanghai Macklin Biochemical Technology Co., Ltd. All experiments were performed between June and December 2024 in controlled laboratory conditions at the Institute of Environment and Sustainable Development in Agriculture, Chinese Academy of Agricultural Sciences.

### 4.2 Experimental design

Uniform, plump okra seeds were surface-sterilized using a 1% sodium hypochlorite solution for 15 min, rinsed thoroughly with deionized water, and soaked at 26°C in darkness for 24 h. The soaking solution was replaced every 12 h. After soaking, the seeds were rinsed three times with deionized water and air-dried under sterile conditions.

Salinity stress conditions were established based on soil salinity classification standards. Four NaCl concentrations were prepared to simulate varying levels of saline stress: 0.1% (2.58 dS/m, 17.1 mM, representing light salinity), 0.3% (7.32 dS/m, 51.3 mM, moderate salinity), 0.5% (11.73 dS/m, 85.5 mM, severe salinity), and 0.7% (15.91 dS/m, 119.8 mM, very severe salinity). Deionized water (16.17 μS/cm) was used as the control. The conductivity of each solution was confirmed using a calibrated conductivity meter to ensure consistent and reproducible salinity levels.

After pre-treatment, seeds were placed on double-layered filter paper in sterilized Petri dishes (50 seeds per dish, three replicates per treatment). Each dish was moistened with the respective NaCl solutions or deionized water (control). The dishes were incubated in a climate-controlled chamber under a 14-h light/10-h dark cycle with a photo flux density of 250 µmol m^–2^ s^–1^ (provided by red and blue LEDs at a 4:1 R:B ratio), 28°C/22°C day/night temperature, and 75% relative humidity. The filter paper and NaCl solutions were replaced daily to maintain consistent salinity and moisture conditions. Germination rate, germination vigor, germination index, and seedling vigor index were recorded at regular intervals. After seven days, the roots and hypocotyls of germinated seedlings were scanned using an Epson Perfection V800 Photo scanner, and their lengths were measured with ImageJ software.

### 4.3 Seedling growth and salt tolerance assessment

Ten seeds of uniform size were selected and transplanted into pots (5 × 5 × 6 cm) filled with autoclaved sand. After germination, seedlings with consistent growth were retained for further sampling and treatment. Salt stress was applied from the germination stage onward, with the pots irrigated with NaCl solutions of varying concentrations (0.1–0.7%), and deionized water serving as the control. The experimental design included five salinity treatments, each with 25 biological replicates. Seedlings were grown under the same controlled environmental conditions used for germination assays. Salinity solutions were applied every five days, ensuring thorough wetting of the substrate. After 16 days of growth, seedlings were sampled at the two-leaf stage for physiological, morphological, and molecular analyses.

### 4.4 Evaluated variables

The following formula was used to determine the total germination for each treatment using germination rate (GR) and germination vigor (GV):


(1) $$GV\left(\%\right)\;\frac{\text{Number of seeds germinated at }2\text{ day}}{\text{Total numberof seeds}}\;100\%$$

(2) $$GR\left(\%\right)\;\frac{\text{Number of seeds germinated at }7\text{ day}}{\text{Total numberof seeds}}\;100\%$$

### 4.5 Growth and morphological traits

At the end of the growth period, seedlings were carefully removed from pots, and roots were gently washed with deionized water. Shoot height was measured with a vernier caliper, and fresh weights of the entire seedling, shoots, and roots were recorded using an analytical balance. Root morphological parameters—including total root length, root surface area, and root diameter—were quantified using the WinRHIZO root analysis system (Regent Instruments, Inc., Québec, QC, Canada) after scanning the roots. All samples were then oven-dried (105°C for 30 min followed by 80°C until constant weight) and reweighed to determine dry biomass.

### 4.6 Chlorophyll fluorescence imaging and photosynthetic efficiency

On day 16, chlorophyll fluorescence parameters were measured using the IMAGING-PAM Chlorophyll Fluorescence System (Walz, Effeltrich, Germany). Parameters included the maximum quantum efficiency of PSII photochemistry (*Fv/Fm*), the effective quantum yield of PSII [Y(II)], as well as the quantum yields of regulated [Y(NPQ)] and non-regulated [Y(NO)] energy dissipation. Additionally, relative electron transport rates (ETR) were quantified to evaluate photosynthetic performance under varying salinity treatments.

### 4.7 Physiological biochemical analysis

The activities of peroxidase (POD), catalase (CAT), superoxide dismutase (SOD), and the level of malondialdehyde (MDA) were determined using the corresponding assay kits (BC0095, Solarbio), following the manufacturer’s instructions. At designated time points, the aerial parts of okra plants were harvested, rapidly frozen in liquid nitrogen, and ground into a fine powder (40 Hz, 1 min). For each sample, 100 mg of the powdered tissue was homogenized in the phosphate-buffered saline (PBS) solution supplied with the kits. The homogenates were vortexed for 10 min, followed by centrifugation at 12,000 rpm for 5 min at 4°C. The resulting supernatants were then used for the enzymatic activity assays, which were conducted on a Thermo Scientific Microplate reader.

### 4.8 RNA isolation and sequencing

Leaf samples from okra plants were collected four hours after treatment with salinity stress. Total RNA was extracted from pooled okra leaves, which were obtained from three independent biological replicates, each consisting of five individual plants. RNA isolation was carried out using the Trizol reagent (Tiangen Biotech, Beijing, China) following the manufacturer’s protocol. For RNA sequencing, libraries were prepared using the NEBNext^®^ Ultra™ RNA Library Prep Kit for Illumina^®^ (NEB, USA) according to the manufacturer’s instructions. Since okra lacks a reference genome, clean reads were processed and assembled *de novo*. Raw reads were first filtered to remove adapters, poly-N stretches, and low-quality reads (Q score< 20). Clean reads were then assembled into contigs using Trinity to construct a reference transcriptome. This assembled transcriptome served as the foundation for downstream analyses, including differential gene expression and functional annotation.

### 4.9 Differential expression analysis and functional annotation

Gene expression levels were quantified based on transcript abundance using methods suitable for *de novo* assemblies, and differential expression analysis was conducted using DESeq2 in R. Genes were considered differentially expressed if they had an adjusted *P*-value (Benjamini-Hochberg correction) of less than 0.05. Functional annotation of these DEGs was performed by BLASTx comparison against protein databases of related species, enabling identification of potential gene functions and biological pathways. To explore the functional significance of these DEGs, gene ontology (GO) enrichment and Kyoto Encyclopedia of Genes and Genomes (KEGG) pathway analyses were conducted using the GOseq R package (v1.22) and KOBAS software (v2.0.12), respectively.

### 4.10 qRT-PCR analysis

Quantitative real-time PCR (qRT-PCR) was conducted to confirm the expression levels of target genes. Total RNA was extracted and reverse-transcribed into cDNA using the PrimeScript™ RT reagent Kit with gDNA Eraser (Thermo Fisher Scientific, USA). qRT-PCR was then performed using TB Green^®^ Premix Ex Taq™ II (Tli RNaseH Plus) (Takara Bio), with primers listed in **Supplementary Table S4**. Relative gene expression was quantified using the 2^−ΔΔCT^ method, as outlined by Livak and Schmittgen (2001).

### 4.11 Statistical analysis

All data were subjected to one-way analysis of variance (ANOVA) using IBM SPSS Statistics 26. Significant differences between treatments were determined at *P*< 0.05. Data visualization, including graphs and tables, was performed using GraphPad Prism 9 and Origin 2025.

## Data Availability

The original contributions presented in the study are publicly available. This data can be found at the National Center for Biotechnology Information (NCBI) using accession number PRJNA1287490.
