# Supplementary material for: Salt gradient-driven adaptation in okra: uncovering mechanisms of tolerance and growth regulation
Source: Front Plant Sci. 2025 Jul 23;16:1648092. doi: 10.3389/fpls.2025.1648092 (PMC12327092; doi:10.3389/fpls.2025.1648092)
Supplement: Supplementary file 1 [file SupplementaryFile1.docx]

Supplementary Material

**Salt gradient-driven adaptation in okra: Uncovering mechanisms of tolerance and growth regulation**

Xi Yang ^1†^, Jiuxing He ^1,2†^, Lifeng Xu ^3†^, Meng Kong ^1^, Qiuyan Huo ^1^, Jiqing Song ^1^, Wei Han ^4*^, and Guohua Lv ^1,5*^

^1^Institute of Environment and Sustainable Development in Agriculture, Chinese Academy of Agricultural Sciences, Beijing 100081, China

^2^Xianghu Laboratory, Hangzhou, China

^3^Mihe National Wetland Park Management Service Center, Qingzhou 262513, China

^4^Shandong Agri-tech Extension Center, Jinan 250013, China

^5^State Key Laboratory of Efficient Utilization of Agricultural Water Resources, CAU/CAAS,

Beijing 100081, China

^†^These authors contributed equally to this work

*** Correspondence:**Corresponding Author
whan01@163.com (W.H.); lvguohua@caas.cn (G.L.)


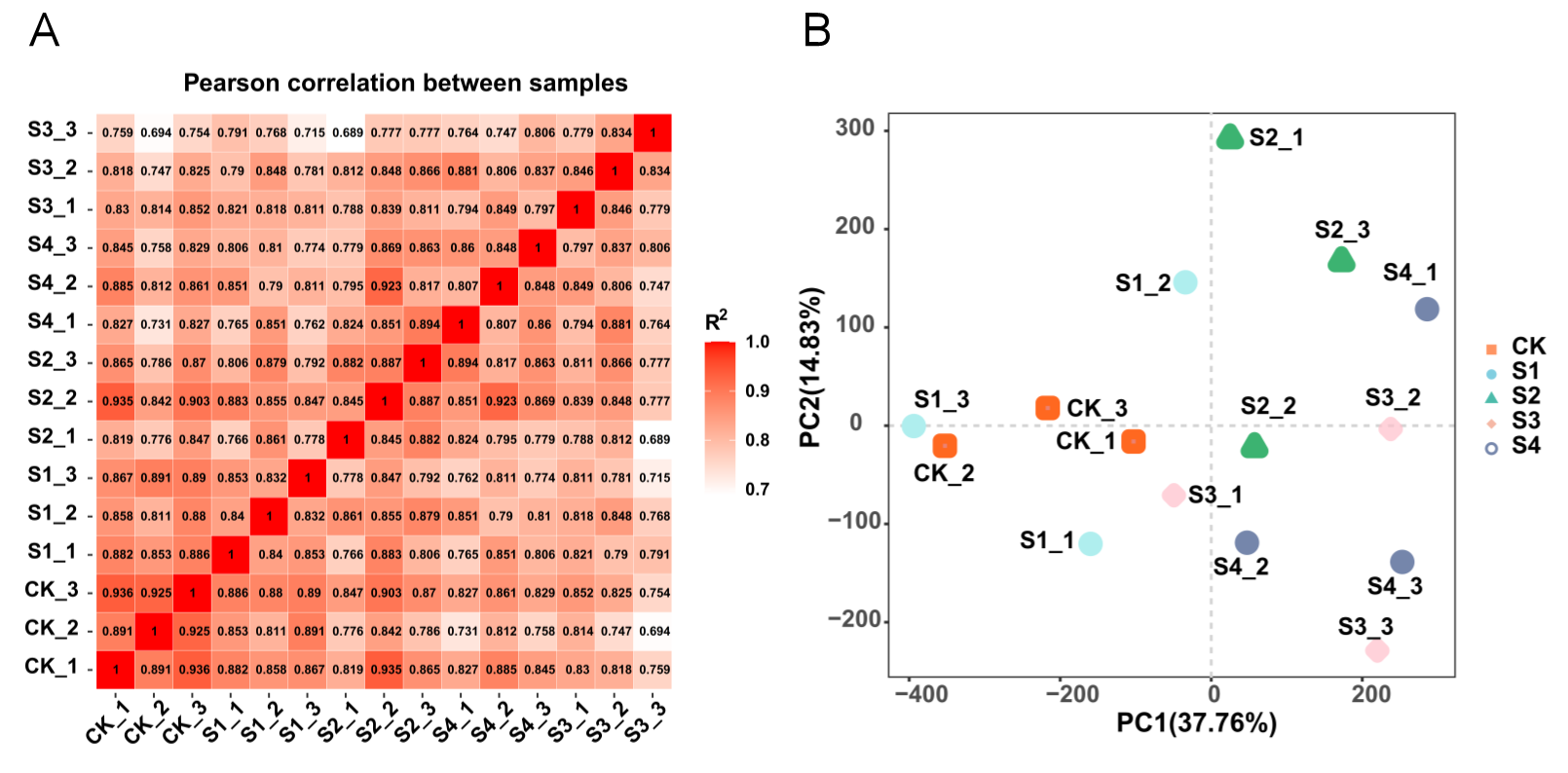


**Supplementary Figure 1.** (A) Hierarchical clustering of samples based on pairwise sample-to-sample distance matrix. (B) Principal component analysis (PCA) of RNA-seq data from okra seedlings subjected to control and salt stress treatments. Sample group abbreviations: CK—0% NaCl (control); S1—0.1% NaCl; S2—0.3% NaCl; S3—0.5% NaCl; S4—0.7% NaCl.

**Supplementary Figure 2.** **Hierarchical clustering analysis of gene expression patterns in okra seedlings under different NaCl treatments.** Okra seeds were continuously exposed to either ddH_2_O (control) or NaCl concentrations ranging from 0.1% to 0.7%, and RNA samples were collected at 16 days of age for RNA-seq analysis. The figure presents (from left to right): gene expression trend fold plots, clustering analysis of differentially expressed genes (DEGs), and GO functional annotation (cellular component, CC). Blue and red shading indicate down-regulated and up-regulated genes, respectively, highlighting significant expression changes in response to salinity stress.

**Supplementary Figure 3.** **Hierarchical clustering analysis of gene expression patterns in okra seedlings under different NaCl treatments.** Okra seeds were continuously exposed to either ddH_2_O (control) or NaCl concentrations ranging from 0.1% to 0.7%, and RNA samples were collected at 16 days of age for RNA-seq analysis. The figure presents (from left to right): gene expression trend fold plots, clustering analysis of differentially expressed genes (DEGs), and GO functional annotation (molecular function, MF). Blue and red shading indicate down-regulated and up-regulated genes, respectively, highlighting significant expression changes in response to salinity stress.


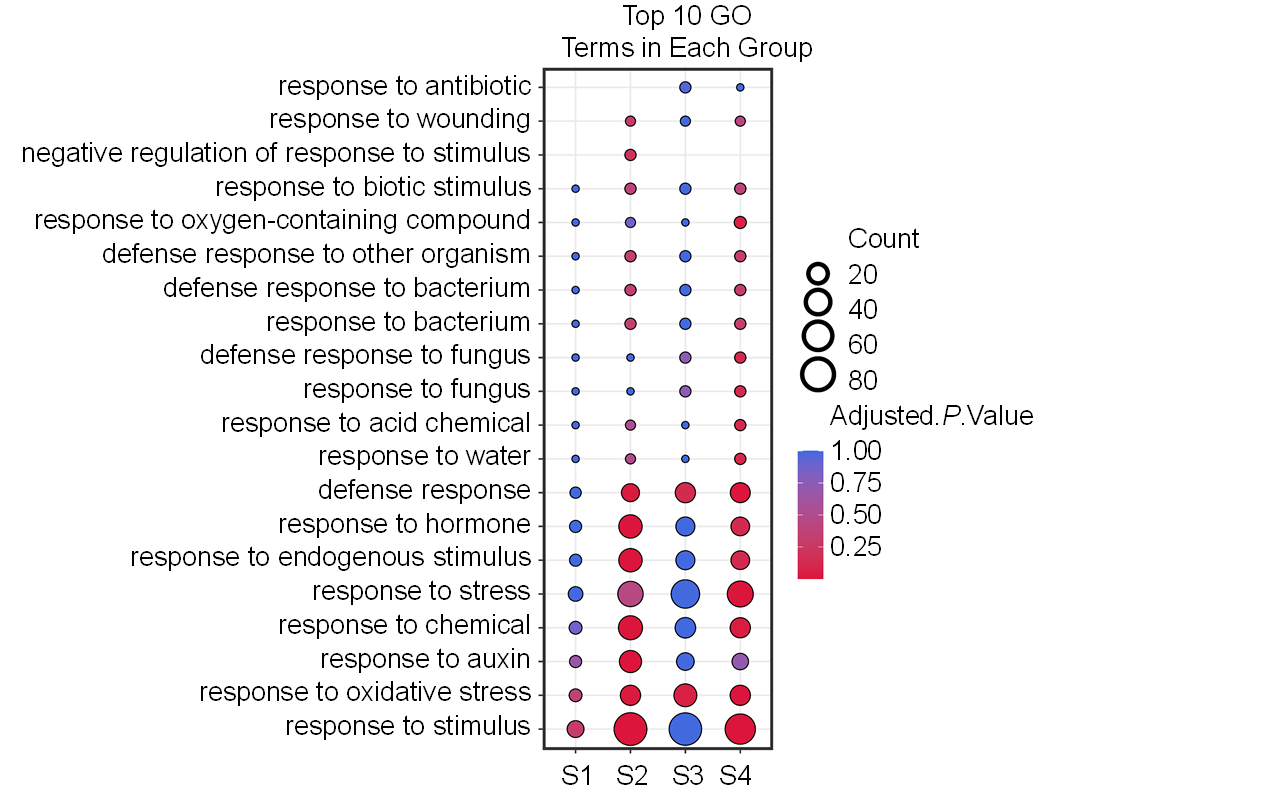


**Supplementary Figure 4.** **Enrichment of environmentally responsive go terms in okra seedlings under varying nacl concentrations.** The bubble chart illustrates the top ten enriched Gene Ontology (GO) terms related to “response” in okra seedlings exposed to varying NaCl concentrations (0%, 0.1%, 0.3%, 0.5%, and 0.7% NaCl). Each circle represents a distinct GO term, with the size of the circle corresponding to the number of genes associated with that term, reflecting the relative significance of each biological process. The color gradient, ranging from blue to red, indicates the adjusted P-values, with red representing terms of higher statistical significance. The analysis specifically highlights the GO terms involved in response mechanisms to salinity stress, with larger circles signifying more extensive gene involvement. Only GO terms with enrichment in more than 3 genes are visualized to ensure clarity and emphasize the most significant biological interactions.
